# Successful Operation for Strangulated Hernia in a Young Lady

**Published:** 1878-01

**Authors:** Henry F. Andrews

**Affiliations:** Physician to St. Joseph’s Orphanage, Washington, Wilkes County, Ga.


ATLANTA
Medical and Surgical Journal

Vol. XV.] JANUARY—1878. [No. 10

### Original Communications.

### SUCCESSFUL OPERATION FOR STRANGULATED HERNIA IN A YOUNG LADY.

By HENRY F. ANDREWS, M.D.,
Physician to St. Joseph's Orphanage, Washington, Wilkes County, Ga.

Although hernia is, by no means, a rare disease, and its relief by an operation of such frequent occurrence as to render it not at all uncommon, yet the following case may not prove altogether uninteresting, on account of at least one feature in it; that is, the occurrence of *inguinal* hernia in a female, and a young lady only sixteen years of age, which is quite unusual. It is a well-established fact that females are much more liable to femoral hernia than males, but not so liable to inguinal.

I give the following entirely from memory with regard to everything except dates. I kept no notes of the case, and can give no minute details, such as frequency of pulse, respiration, etc., but the facts of any importance are all correctly given.

On Wednesday, January 21, 1876, about ten o'clock A.M., I received a note from Dr. J. S. Lane, requesting me to meet him and Dr. H. Q. Harper in consultation, at the residence of Mr. C., some nine miles from this place, and in the village of Rehoboth. The note also stated that the daughter of Mr. C. was suffering with strangulated hernia, and contained the request that I bring instruments and everything necessary, and come prepared to operate.

On reaching the house, about twelve o'clock M., I found the patient to be a young lady sixteen years of age, and suffering as the note stated, with strangulated hernia. The tumor was upon the right side, and proved to be oblique inguinal hernia.

The disease was of about one year's standing, and she had been accustomed to the wearing of a truss, but this becoming too weak or too small from her growing, failed to support the part properly, and to keep the intestine from descending, at times.

On Monday morning the 19th, while at school, the intestine came down and caused such intense pain that she fainted. She was taken home, about half a mile distant, and the family physician, Dr. J. S. Lane, sent for. On arriving, he endeavored to reduce the hernia, but failed. He persisted in his endeavors to reduce it by taxis, and gave chloroform, used the warm bath, and nauseants, and tried everything recommended in such cases. He remained with her all day, and through the night, and then sent for Dr. H. Q. Harper, a neighboring physician. On his arrival, efforts to reduce were renewed, and taxis, aided by all the usual adjuvants employed in such cases, was persisted in without avail. There was excessive pain and the efforts to reduce caused great aggravation of it, so that anodynes—especially opiates—were freely given. These two gentlemen remained all Tuesday night, and on Wednesday morning sent to this place for consultation.

On hearing the statement of Drs. Lane and Harper, I was satisfied that an operation would be necessary as the only means of relief, and upon a careful examination of the tumor and an effort to reduce it, I was more thoroughly convinced. This conclusion had already been reached by both the other physicians, and the nature of the case was carefully explained to the parents, and also to the patient. I stated to them that we would put her fully under the influence of chloroform once more, and use every means to reduce the hernia, but, in case we still failed, we had determined that there was no other hope of her recovery except by the knife, and that I would, in that event, operate without allowing her to come from under the influence of the anæsthetic. The consent of all interested having been obtained to proceed as we deemed best, she was immediately brought under chloroform by Dr. Lane, which she yielded to in a short time, and in a most pleasant manner. Taxis was again tried faithfully, but with the same result as before. There was a peculiarly firm, obstinate kind of sensation experienced on pressing upon the hernia, which gave me the impression, at the first touch, that it could not be reduced except by an operation. After we had tried to reduce it in every way we could, and failing, I took the knife and made an incision through the integument over the tumor. I then divided the layers carefully, without regard to the number of coverings, which I think is utterly useless, if not impossible to be done upon the living subject. The rule which I regard as the one of greatest importance is, to cut until you come to the sack—the only important point being to know the sack when you reach it, and to be able to distinguish between sack and bowel. Of course this cutting must be done with great care upon the grooved directory, each layer being taken up and divided separately till the sack is reached, and it is of no importance whatever, whether four or forty layers are taken up and divided.

On reaching the sack it was readily recognized by the arborescent appearance of the blood-vessels, and was opened, when a small quantity of serous fluid—some two or three drachms—escaped, and the bowel was brought clearly into view. This was found to be congested, but there was no evidence of gangrene. The stricture being within the sack, I passed my finger up to the external ring, where there existed a very tight stricture, through which it was very difficult to pass the point of the finger nail and to introduce the point of a probe-pointed curved bistoury (I had no hernia knife with me, though I was not aware of the fact till I went to prepare for the operation). After introducing the bistoury, I cut, according to rule, directly upwards, nicking the stricture, which gave way, admitting the bowels readily, when I withdrew the knife.

The intestine was returned to the cavity ef the abdomen, the edges of the wound drawn together, and secured by several points of interrupted sutures, the parts thoroughly cleansed, the sutures supported by adhesive strips, a compress of linen laid over the wound and secured by several turns of a spica bandage, when she was allowed to come from under the chloroform.

She complained of a pain in the abdomen and was quite restlless. I injected one-fourth of a grain of sulphate of morphia in the arm, which relieved the pain considerably, and caused her to rest more quietly.

I left her some two hours after the operation, at which time she was doing very well, pulse good and everything indicating as fair a prospect for recovery as could be expected. I visited her again on the 23d, meeting Drs. Lane and Harper. She was doing remarkably well. Her bowels had acted the day previous. There was considerable soreness in the region of the wound and some pain in the abdomen; pulse about 100. She had some appetite, and was allowed fluid nourishment. There was some little swelling and tympanitis over the abdomen; tongue clean. I directed the discontinuance of all opiates unless the pain became so severe as to render their use absolutely necessary. The wound seemed to be doing well, and I did not disturb it. I also directed the administration of a warm water enema, with soap and spirit of turpentine, in case the bowels did not move by the next morning, as I was satisfied the action she had the day before did not come from above the strictured portion of the bowel, but was merely a discharge of some fecal masses which had been lying in the intestine below the point. I did not deem it prudent to give a purgative in the condition the intestine must have been in about the strictured point, but preferred to empty the bowels by enema, which I considered incapable of doing any injury.

On the 25th I again visited the patient, meeting the above named physicians again. Found her getting on remarkably well, but complaining of some uneasiness just at the seat of the wound. Her bowels had been moved freely by means of the enema, relieving the swelling and tympanitis entirely, and causing her to feel decidedly more comfortable. Her appetite was now good, her pulse natural, tongue clear, and condition in every way as favorable as possible. Removed the dressings from the wound, which was found to be united except at the points where the sutures passed. At these points there was a discharge of pus. The sutures were all removed, and, after washing the parts well with warm water, adhesive strips were applied, the compress replaced and the spica bandage again applied. From this time she rapidly recovered without a single unfavorable symptom, my next and last visit being on the 28th of June, when recovery was so far advanced and so well assured that I deemed any further attention on my part unnecessary.

Operations for strangulated hernia are deemed amongst the most dangerous and most fatal of surgical operations. This fatality is attributed, in great a measure, to the fact that these operations are almost invariably postponed until too late, or until they are performed merely as a last resort, and as almost hopeless. The consequence is, as we have seen it, that when the sack is opened the bowel is found either in a state of gangrene or so near it that recovery is almost impossible or almost hopeless. In the above case, Drs. Lane and Harper showed their sound judgment in deciding that an operation was necessary before the case became hopeless, and in taking steps to have it performed at once, after all other means of relief had been faithfully tried and had failed, and after waiting as long as it was prudent to wait for relief to come by any effort of nature. When I reached her the first unfavorable symptoms were beginning to manifest themselves. Her pulse was becoming accelerated, she was becoming restless and nervous, and her face began to wear an anxious look, characteristic of approaching trouble of a serious nature. If the operation had been postponed until next day, or even for a few hours, the result would have been at least more doubtful.

In conclusion, I would say that Drs. Lane and Harper not only exhibited consummate skill and judgment in their management of the case, but rendered me invaluable assistance in the performance of the operation.

